# Cortisol awakening response in bipolar patients with comorbid type 2 diabetes mellitus

**DOI:** 10.1192/j.eurpsy.2024.674

**Published:** 2024-08-27

**Authors:** M. Battipaglia, N. Attianese, S. Donato, R. Ceres, R. Cerra, A. M. Monteleone, P. Monteleone, G. Cascino

**Affiliations:** ^1^University of Campania “L.Vanvitelli”, Naples; ^2^University of Salerno, Salerno, Italy

## Abstract

**Introduction:**

Bipolar Disorder (BD) is a severely debilitating psychiatric disorder with high rates of morbidity and mortality, and patients with BD have a 10-year reduction in their life expectancy. Bipolar disorder (BD) is frequently associated with type 2 diabetes mellitus (T2DM). BD patients with comorbid T2DM have been shown to have three times higher odds of a chronic course and rapid cycling and are more likely to present worse outcomes to treatment with lithium and/or other mood stabilisers when compared to BD patients without IGM (impaired glucose metabolism).

**Objectives:**

The functioning of the hypothalamic-pituitary-adrenal (HPA) axis has been never investigated in BD with respect to the glucose metabolic status. Therefore, we assessed the cortisol awakening response (CAR) in bipolar patients with or without comorbid T2DM.

**Methods:**

Twenty euglycemic bipolar patients [12 males and eight females; mean age (±SD): 47.4 ± 14.4 years; mean (±SD) duration of illness: 18.3 ± 12.1 years], 16 BD patients with T2DM [11 males and five females; mean age (±SD): 63.6 ± 12.8 years; mean (±SD) duration of bipolar illness: 17.1 ± 10.8 years; mean (±SD) duration of T2DM: 5.2 ± 5.3 years], 18 healthy subjects [seven males and 11 females; mean age (±SD): 45.0 ± 12.1 years] and 12 non-psychiatric subjects with T2DM [eight males and four females; mean age (±SD): 56.7 ± 11.2 years; mean (±SD) duration of T2DM: 5.2 ± 3.5 years] were recruited. Saliva cortisol was measured at awakening and after 15, 30, and 60 min.

**Results:**

With respect to both healthy controls and controls with T2DM, euglycemic and diabetic BD patients exhibited a CAR occurring at significantly lower levels. No significant difference emerged in the CAR between the two groups of bipolar patients. Controls with T2DM had an overall post-awakening cortisol production significantly higher than healthy controls.

**Conclusions:**

Our results show that the CAR of patients with BD is reduced in terms of overall cortisol production but normal in terms of cortisolo reactivity independently from the occurrence of comorbid T2DM. The dampened CAR points to a tuning down of the functioning of the HPA axis in both euglycemic and diabetic BD patients, which may be a factor of vulnerability, since a preserved HPA axis functioning is essential to deal with stressors, which may precipitate affective episodes

**Disclosure of Interest:**

None Declared

